# Textbook outcome in urgent early cholecystectomy for acute calculous cholecystitis: results post hoc of the S.P.Ri.M.A.C.C study

**DOI:** 10.1186/s13017-024-00539-6

**Published:** 2024-03-21

**Authors:** Paola Fugazzola, Silvia Carbonell-Morote, Lorenzo Cobianchi, Federico Coccolini, Juan Jesús Rubio-García, Massimo Sartelli, Walter Biffl, Fausto Catena, Luca Ansaloni, Jose Manuel Ramia, the S.P.Ri.M.A.C.C. Collaborative Group, Trpimir Morić, Selmy Awad, Azzah M. Alzahrani, Mohamed Elbahnasawy, Damien Massalou, Belinda De Simone, Zaza Demetrashvili, Athina‑Despoina Kimpizi, Dimitrios Schizas, Dimitrios Balalis, Nikolaos Tasis, Maria Papadoliopoulou, Petrakis Georgios, Konstantinos Lasithiotakis, Orestis Ioannidis, Lovenish Bains, Matteo Magnoli, Pasquale Cianci, Nunzia Ivana Conversano, Alessandro Pasculli, Jacopo Andreuccetti, Elisa Arici, Giusto Pignata, Guido A. M. Tiberio, Mauro Podda, Cristina Murru, Massimiliano Veroux, Costanza Distefano, Danilo Centonze, Francesco Favi, Vanni Agnoletti, Rafaele Bova, Girolamo Convertini, Andrea Balla, Diego Sasia, Giorgio Giraudo, Anania Gabriele, Nicola Tartaglia, Giovanna Pavone, Fabrizio D’Acapito, Nicolò Fabbri, Francesco Ferrara, Stefania Cimbanassi, Luca Ferrario, Stefano Ciof, Marco Ceresoli, Chiara Fumagalli, Luca Degrate, Maurizio Degiuli, Silvia Sofa, Leo Licari, Matteo Tomasoni, Tommaso Dominioni, Camilla Nikita Farè, Marcello Maestri, Jacopo Viganò, Benedetta Sargenti, Andrea Anderloni, Valeria Musella, Simone Frassini, Giulia Gambini, Mario Improta, Alberto Patriti, Diego Coletta, Luigi Conti, Michele Malerba, Muratore Andrea, Marcello Calabrò, Beatrice De Zolt, Gabriele Bellio, Alessio Giordano, Davide Luppi, Carlo Corbellini, Gianluca Matteo Sampietro, Chiara Marafante, Stefano Rossi, Andrea Mingoli, Pierfrancesco Lapolla, Pierfranco M. Cicerchia, Leandro Siragusa, Michele Grande, Claudio Arcudi, Amedeo Antonelli, Danilo Vinci, Ciro De Martino, Mariano Fortunato Armellino, Enrica Bisogno, Diego Visconti, Mauro Santarelli, Elena Montanari, Alan Biloslavo, Paola Germani, Claudia Zaghi, Naoki Oka, Mohd Azem Fathi, Daniel Ríos‑Cruz, Edgard Efren Lozada Hernandez, Ibrahim Umar Garzali, Liliana Duarte, Ionut Negoi, Andrey Litvin, Sharfuddin Chowdhury, Salem M. Alshahrani, Silvia Carbonell‑Morote, Juan J. Rubio‑Garcia, Claudia Cristina Lopes Moreira, Iñigo Augusto Ponce, Fernando Mendoza‑Moreno, Anna Muñoz Campaña, Heura Llaquet Bayo, Andrea Campos Serra, Aitor Landaluce, Begoña Estraviz‑Mateos, Izaskun Markinez‑Gordobil, Mario Serradilla‑Martín, Antonio Cano‑ Paredero, Miguel Ángel Dobón‑Rascón, Hytham Hamid, Oussama Baraket, Emre Gonullu, Sezai Leventoglu, Yilmaz Turk, Çağrı Büyükkasap, Ulaş Aday, Yasin Kara, Hamit Ahmet Kabuli, Semra Demirli Atici, Elif Colak, Serge Chooklin, Serhii Chuklin, Federico Ruta, Marcello Di Martino, Francesca Dal Mas, Fikri M. Abu‑Zidan, Salomone Di Saverio, Ari Leppäniemi, Elena Martín‑Pérez, Ángela de la Hoz Rodríguez, Ernest E. Moore, Andrew B. Peitzman

**Affiliations:** 1 Division of General Surgery, Fondazione IRCCS Policlinico San Matteo, Pavia, Italy; 2 Servicio de Cirugía General. Hospital General Universitario Dr. Balmis, Alicante, Spain; 3 ISABIAL: Instituto de Investigación Sanitaria y Biomédica, Alicante, Spain; 4 Department of Clinical, Diagnostic and Pediatric Sciences, University of Pavia, Via Alessandro Brambilla, 74, 27100 Pavia, PV Italy; 5 Department of Emergency and Trauma Surgery, Pisa University Hospital, University of Pisa, Pisa, Italy; 6 Macerata Hospital, 62100 Macerata, Italy; 7 Gastroenterology and Digestive Endoscopy Unit, Fondazione IRCCS Policlinico San Matteo, Pavia, Italy; 8 Division of Trauma/Acute Care Surgery, Scripps Clinic Medical Group, La Jolla, CA USA; 9 General and Emergency Surgery, Bufalini Hospital, Cesena, Italy; 10 Department of Pathology. and Surgery, Universidad Miguel Hernandez, Ctra Valencia 23C, 03550 Sant Joan d´Alacant, Spain

**Keywords:** Textbook outcome, Benchmark, Early cholecystectomy, Acute cholecystitis, Morbidity

## Abstract

**Introduction:**

A textbook outcome patient is one in which the operative course passes uneventful, without complications, readmission or mortality. There is a lack of publications in terms of TO on acute cholecystitis.

**Objetive:**

The objective of this study is to analyze the achievement of TO in patients with urgent early cholecystectomy (UEC) for Acute Cholecystitis. and to identify which factors are related to achieving TO.

**Materials and methods:**

This is a post hoc study of the SPRiMACC study. It´s a prospective multicenter observational study run by WSES. The criteria to define TO in urgent early cholecystectomy (TOUEC) were no 30-day mortality, no 30-day postoperative complications, no readmission within 30 days, and hospital stay ≤ 7 days (75th percentile), and full laparoscopic surgery. Patients who met all these conditions were taken as presenting a TOUEC.

**Outcomes:**

1246 urgent early cholecystectomies for ACC were included. In all, 789 patients (63.3%) achieved all TOUEC parameters, while 457 (36.6%) failed to achieve one or more parameters and were considered non-TOUEC. The patients who achieved TOUEC were younger had significantly lower scores on all the risk scales analyzed. In the serological tests, TOUEC patients had lower values for in a lot of variables than non-TOUEC patients. The TOUEC group had lower rates of complicated cholecystitis. Considering operative time, a shorter duration was also associated with a higher probability of reaching TOUEC.

**Conclusion:**

Knowledge of the factors that influence the TOUEC can allow us to improve our results in terms of textbook outcome.

## Introduction

Acute cholecystitis (ACC) is a very common pathology, and accounts for between 3 and 7% of the causes of urgent consultation for abdominal pain [1–3]. The morbidity rate in surgical treatment of ACC ranges between 7.2 and 26%, and the mortality rate between 0 and 10%. Due to the high prevalence of the condition, reducing the post-operatve morbidity and mortality is a priority issue and would have a great impact in this area of health management [2].

The high variability in the morbidity and mortality figures are due to several factors. Some of them are patient-specific such as age and associated comorbidities, the duration of the condition, and its form of presentation (i.e., associated with liver abscess, perforated, gangrenous, emphysematous, etc.). Mortality associated with ACC is especially high in elderly patients, with associated cardiovascular comorbidity and with complicated forms of the disease [2].

The management and treatment of ACC has been standardized in recent years with the publication of the Tokyo Guidelines in 2013 and 2018, and the WSES in 2020 [1, 5, 6]. According to these guidelines, the therapeutic decision depends on the general condition of the patient and the time of evolution of the clinical picture [1, 7]. Early laparoscopic cholecystectomy (ELC, i.e., within 72 h of the onset of symptoms), in the absence of severity criteria that contraindicate it, constitutes the gold standard for the current management of ACC [1, 5, 6, 8].Since ELC seems significantly reduce intra-operative laparoscopic conversion to open, bile duct injury and post-operative length of stay (LOS) and a significantly greater proportion of ELC is undertaken in high-volume centres, it could be suggested that if ACC is operated on exclusively by high-volume emergency laparoscopist surgeons, the results obtained could be improved[4].

Textbook outcome (TO) is a multidimensional measure used to assess the quality of surgical practice. It reflects an "ideal" surgical result, based on a series of benchmarks or established reference points that may vary depending on the pathology [9]. The first time this management tool was mentioned in the literature was in 2013, when Kolfschoten et al. defined eight parameters that characterized TO in colorectal cancer surgery [9]. Since then, numerous publications have emerged defining TO in other areas of cancer surgery (e.g., pancreatic, hepatobiliary, esophagogastric surgery, etc.) [10–14].

References to TO in the literature in benign diseases are scarce. The few reports that are available were all published very recently [15–17]. In the case of ACC, there is no established consensus regarding the parameters that should be included in the definition of TO [17, 18]. There is only one article that defines TO in acute cholecystitis [18], and one that defines it in scheduled laparoscopic cholecystectomy [17]. Based on these two manuscripts, the items for achieving TO in urgent early cholecystectomy (UEC) include no Clavien-Dindo complication (< I), hospital stay less than the 75th percentile, no mortality or readmission within 30 days of surgery and the laparoscopic approach. All patients who presented these variables were considered TO in UEC [9].

## Objective

The primary endpoint was to identify factors related to achieving TO in patients with urgent early cholecystectomy (UEC) for ACC. Secondary objectives were to provide an international proposal for defining the parameters for defining TO in the surgical treatment of this condition.

## Methods

The SPRiMACC study is a prospective multicenter observational study run by the World Society of Emergency Surgery (WSES). From 1 September 2021 to 1 September 2022, consecutive patients admitted to 79 centers located in 19 different countries were included. The study was registered on ClinicalTrial.gov with the following identifier: NCT04995380 [19].

Inclusion criteria were: 1: a diagnosis of ACC as defined by 2018 TG criteria, 2: being a candidate for UEC during the index admission (other surgical techniques, either open or bailout procedures such as subtotal cholecystectomy, were not reasons for intraoperative exclusion), 3: age ≥ 18 years old, 4: being stratified for the risk of common bile duct stones, and, if confirmed, reception of preoperative ERCP, 5: providing a signed and dated informed consent form, and 6: willingness to comply with all study procedures, and being available for the duration of the study.

Exclusion criteria were 1: pregnancy or lactation, 2: acute cholecystitis not related to a gallstone etiology, 3: onset of symptoms > 10 days before cholecystectomy (patients with ACC associated with common bile duct stones who underwent preoperative ERCP were included if they had received EC within 10 days of onset of symptoms), 4: concomitant cholangitis or pancreatitis, 5: intraoperative treatment of common bile duct stones, or 6: any other factors that might increase the risk for the patient or preclude their full compliance with the execution of the study.

The following items were analysed: gender, age, body mass index (BMI), and the following scores: POSSUM Physiological Score [20, 21], APACHE II [22], ASA [23], Charlson Comorbidity Index [24], and modified frailty index [25]; clinical data: pulse (rate per minute), systolic blood pressure (mmHg), temperature (°C degrees); hypertension requiring treatment, diabetes mellitus treated with insulin or oral medication, liver disease, previous abdominal surgical procedures, time since symptoms and surgery (days), duration of symptoms > 72 h, palpable tender mass in the right upper abdominal quadrant, leukocytes > 18,000/mm^3^, hemoglobin (gr/dl), platelet count, INR, creatinine, bilirubin; data regarding ACC: previous percutaneous cholecystostomy, common bile duct stones confirmed by EUS or MRI, gangrenous cholecystitis, pericholecystic abscess, hepatic abscess, biliary peritonitis, ACC grade according to TG guidelines [1, 6] operative time (minutes), bail-out procedure, Chole-Risk score [26], POSSUM Operative Risk Score [20], postoperative complications measured by Clavien-Dindo score at 30 days [27], readmission at 30 days and hospital stay. Complications with Clavien Dindo score CD ≤ II were considered minor, and those with CD ≥ IIIa major.

The criteria used to define TO in urgent early cholecystectomy (TOUEC) were no 30-day mortality, no 30-day postoperative complications (any CD ≥ I is considered non-TO), no readmission within 30 days, and hospital stay ≤ 7 days (75th percentile), and full laparoscopic surgery. The cholecystectomies performed through an initial open approach or with conversion after initial laparoscopy were considered non-TO. Patients who cumulatively presented all the characteristics listed were considered to be TOUEC.

The characteristics of the TO and non-TO groups were compared using IBM® SPSS version 25.0 (SPSS Inc). Continuous variables without normal distribution were expressed as medians with interquartile range (IQR), using the Mann–Whitney U test. Categorical variables were reported as frequencies and proportions and compared using the χ2 test. Subsequently, univariate and multivariate logistic regressions were performed to identify the independent factors associated with obtaining TO. A p < 0.05 was considered statistically significant.

## Results

A total of 1253 patients were studied, but seven were excluded due to incomplete data for analysis. Therefore, 1246 urgent early cholecystectomies for ACC were included. In all, 789 patients (63.3%) achieved all TOUEC parameters, while 457 (36.6%) failed to achieve one or more parameters and were considered non-TOUEC. The parameter with the most impact on achieving TOUEC was the existence of complications, followed by length of stay, laparoscopic approach and 30-day readmission; the one with the least impact on TOUEC was mortality.

**Fig. 1:**
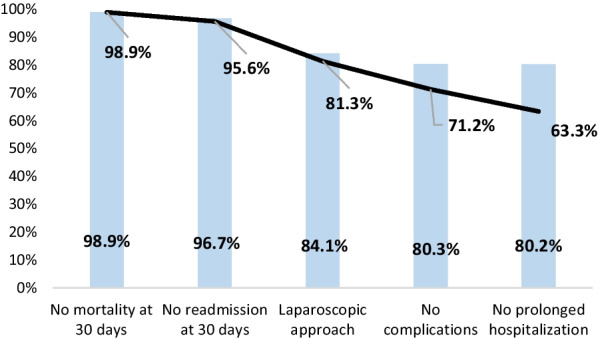
Textbook outcome in emergent cholecystectomy due to acute cholecystitis

Complications at 30 days were recorded in 209 patients (16.7%), meaning that 1037 patients (83.22%) did not present complications at this time point. The complications were minor (CD < II) in 123 patients (9.9%) and major (CD ≥ IIIa) also in 83 patients (6.6%). The surgical approach was laparoscopic in 1048 patients (84.1%), and open in the remaining 15.9%. Fourteen patients had died at 30 days, a mortality rate of 1.1%. Forty-one patients (3.3%) were readmitted at 30 days. The data for achieving TO are shown in Fig. 1, in which each column represents a TOUEC parameter and the blue line shows the cumulative incidence of TOUEC.

Comparison of the TO and non-TO groups revealed several significant differences. The patients that achieved TOUEC were a median of 11 years younger and were more frequently female. TOUEC patients had significantly lower scores (p.000) on all the risk scales analysed (ASA, POSSUM Physiological Score. Charlson Score, Frailty Score, Chole-Risk score, and POSSUM Operative score). Temperature and pulse were also significantly lower in the TOUEC group. Patients with diabetes, hypertension, or heart, liver, and lung diseases were less likely to achieve TOUEC. Prior abdominal surgeries and BMI did not show differences between groups. In the serological tests, TOUEC patients had lower values for creatinine, sodium, potassium, INR, bilirubin, and leukocytosis than non-TOUEC patients.

Regarding the characteristics of the disease itself, the TOUEC group had lower rates of complicated cholecystitis (gangrenous, liver abscess, biliary peritonitis, choledocholithiasis, and emphysematous cholecystitis). Preoperative percutaneous cholecystostomy was less frequently performed in the patients who later emerged as TOUEC. As for surgical time, a shorter duration was also associated with a greater likelihood of achieving TOUEC (see Table 1).

**Table 1:** Characteristics of patients who achieve TO versus non-TO

	No TO	TO	*p* value
n = 1246	n = 457	n = 789	
Gender			**.000**
Male	275 (60.6)	382 (48.9)	
Female	179(39.4)	399 (51.1)	
Age (median IQR)	68 (54–77)	57 (44–70)	**.000**
*BMI (body mass index)	27 (24–29)	27 (24–29)	.929
*Scores*			
*POSSUM Physiological score	22 (18–28)	18 (15–23)	.000
*TOTAL POSSUM	32(27–38)	28 (24–32)	**.000**
*APACHE II SCORE	7(5–11)	5(2–8)	.000
ASA score			**.000**
1	51 (11.9)	214 (29.3)	
2	171 (40)	355 (48.6)	
3	164 (38.3)	152 (20.8)	
4	39 (9.1)	9 (1.2)	
5	3 (0.7)	0 (0)	
Charlson Comorbidity Score > 6			**.000**
No	381 (86.4)	735 (96.6)	
Yes	60 (13.6)	26 (3.4)	
Modified Frailty index			.000
0	150 (32.8)	429 (54.4)	
1	126 (27.6)	176 (22.3)	
2	79 (17.3)	86 (10.9)	
3	47 (10.3)	43 (5.4)	
4	22( 4.8)	19 (2.4)	
5	13 (2.8)	7 (0.9)	
6	7 (1.5)	1 (0.1)	
7	4 (0.9)	1 (0.1)	
8	1 (0.2)	0 (0)	
Clinical data			
*Pulse (rate per minute)	85(76–95)	80(72–90)	**.000**
*Systolic blood pressure (mmHg)	130 (120–145)	130 (120–142)	.432
*Temperature °C	36.9(36.2–37.8)	36.7 (36.1–37.2)	**.001**
Hypertension requiring treatment			**.000**
No	208 (46.1)	243 (53.9)	
Yes	510 (65.6)	267 (34.4)	
Diabetes mellitus treated with insulin or oral medications			**.000**
No	339 (75.3)	662 (85.2)	
Yes	111 (24.7)	115 (14.8)	
Any liver disease			**.024**
No	163 (91.6)	714 (95.7)	
Yes	15 (8.4)	32 (4.3)	
Previous abdominal surgical procedures?			.046
No	309 (70.2)	579 (75.5)	
Yes	131 (29.8)	188 (24.5)	
*Time since symptoms and surgery (days)	4(2–6)	3(1–5)	.000
Duration complaints > 72h			.000
No	245 (53.6)	523 (66.3)	
Yes	211 (46.2)	264 (33.5)	
Palpable tender mass in the right upper abdominal quadrant			**.000**
No	269 (58.9)	539 (68.8)	
Yes	188 (41.1)	245 (31.3)	
Serological tests			
White blood cells > 18.000/mm3			**.000**
No	325 (71.4)	670 (85.5)	
Yes	130 (28.6)	114 (14.5)	
*Hemoglobin	13.2 (11.8–14.6)	13.8 (12.4–15)	**.000**
Platelet count > 100,000/mm3			**.001**
No	436 (95.4)	775 (98.2)	
Yes	20 (4.4)	11 (1.4)	
INR > 1.5			**.000**
No	397 (86.9)	762 (96.6)	
Yes	58 (12.7)	23 (2.9)	
Creatinine > 2mg/dL			**.000**
No	396 (86.7)	769 (97.5)	
Yes	60 (13.1)	19 (2.4)	
Increased total bilirubin > 2 mg/dL			**.000**
No	318 (72.4)	679 (88.8)	
Yes	121 (27.6)	86 (11.2)	
Data About AC			
Previous percutaneous cholecystostomy			**.000**
No	419 (94.8)	758 (99.1)	
Yes	23 (5.2)	7 (0.9)	
Associated Common Bile Duct Stones (confirmed by EUS or MRI)			**.000**
No	393 (86.0)	736 (93.3)	
Yes	45 (9.8)	23 (2.9)	
Gangrenous cholecystitis			**.000**
No	278 (61)	632 (80.2)	
Yes	178 (39)	156 (19.8)	
Pericholecystic abscess			**.000**
No	353 (77.9)	733 (93.3)	
Yes	100 (22.1)	53 (6.7)	
Hepatic abscess			**.000**
No	429 (94.1)	777 (99.4)	
Yes	27 (5.9)	5 (0.6)	
Biliary peritonitis			**.000**
No	419 (92.3)	764 (97.7)	
Yes	35 (7.7)	18 (2.3)	
ACC grade			**.000**
1	98 (21.4)	304 (38.5)	
2	353 (77.2)	485 (61.5)	
3	6 (1.3)	0 (0)	
*Operative time in min	110(85–140)	85(60–116)	.000
Bail-out procedure?			**.000**
No	374 (82)	761 (96.8)	
Yes	82 (18)	25 (3.2)	
Chole-Risk score			.000
0	58 (12.7)	165 (20.9)	
1	182 (39.8)	370 (46.9)	
2	136 (29.8)	187 (23.7)	
3	65 (14.2)	28 (3.5)	
4	9 (2.0)	1 (0.1)	
POSSUM Operative severity score			.000
< 15	390 (85.3)	705 (89.4)	
> 15	47 (10.3)	7 (0.9)	

*BMI* Body mass index, *EUS* endoscopic ultrasound, *MRI* magnetic resonance, *ACC* Acute cholecystitis

^*^Median and interquartilic rank IQR

The results of the univariate logistic regression showed significant differences in numerous variables. Younger age was a protective factor for achieving TOUEC, while female sex increased the possibility of obtaining TOUEC by 1.6 times. However, neither parameter reached significance in the multivariate regression model. Table 2 shows the results of the logistic regression.

**Table 2:** Univariate and multivariate analysis of factors related to achieve to

		Univariant analysis				Multivariant analysis		
	OR	IC95% inf	IC 95% sup	*p* value	OR	IC95% inf	IC 95% sup	*p* value
Gender								
Male (ref) Female	1.605	1.269	2.029	.001	–	–	–	–
Age	0.969	0.962	0.977	.001	–	–	–	–
Scores								
POSSUM Physiological score	.909	.891	.928	.000	.866	.770	.974	.016
Total POSSUM	.912	.896	.929	.000	1.120	.999	1.256	.051
APACHE II SCORE	.855	.826	.884	.000	–	–	–	–
ASA score 1 (ref.) 2 3 4 5	– .495 .221 .055 .000	– .347 .152 .025 .000	– .706 .322 .121 –	– .000 .000 .999 .999	– 171,579,770.904 99,261,356.640 53,879,330.972 26,142,808.327	– .000 .000 .000 .000	– – – – –	.05 – .999 .999 .999 1.000
Charlson Comorbidity Score > 6 No Yes (ref.)	4.452	2.764	7.170	.000	–	–	–	–
Modified Frailty index 0 (ref) 1 2 3 4 5 6 7 8	– .488 .381 .320 .302 .188 .050 .087 .000	– .364 .266 .203 .159 .074 .006 .010 .000	– .656 .544 .503 .573 .481 .409 .788 –	.000 – .000 .000 .000 .000 .000 .005 .030 1.000	–	–	–	–
Clinical data								
Pulse (rpm)	0.974	.966	.982	.000	0.978	0.965	0.992	.002
Temperature °C	1.004	.987	1.020	.667	–	–	–	–
Hypertension requiring treatment	2.232	1.761	2.828	.000	2.157	1.415	3.286	.001
DM treated with insulin or oral medications No Yes (ref.)	1.885	1.408	2.524	.000	–	–	–	–
Any liver disease No Yes (ref.)	2.053	1.087	3.880	.027	–	–	–	–
Time since beginning symptoms and surgery (days)	1.002	.999	1.006	.256	–	–	–	–
Duration complaints > 72h No Yes (ref.)	1.706	1.347	2.161	.000	–	–	–	–
Palpable tender mass in the right upper abdominal quadrant No Yes (ref.)	1.538	1.210	1.954	.000	1.538	1.21	1.954	.001
Oliguria No Yes (ref.)	5.4	3.114	9.366	.000	8.319	2.253	30.714	.001
Serological tests								
White blood cells > 18.000/mm3 No Yes (ref.)	2.351	1.769	3.124	.000	–	–	–	–
Hemoglobin	1.004	.999	1.009	.132	–	–	–	–
Platelet count > 100,000/mm3 No Yes ref	3.232	1.534	6.808	.002	–	–	–	–
INR > 1.5 No Yes (ref.)	4.840	2.942	7.964	.000	–	–	–	–
Creatinine > 2mg/dL No Yes (ref.)	6.132	3.610	10.419	.000	4.331	1.272	14.748	.019
Increased total bilirubin > 2 mg/dL? No Yes (ref.)	3.004	2.210	4.083	.000	–	–	–	–
Data about AC								
Previous percutaneous cholecystostomy No Yes (ref.)	5.944	2.529	13.969	.000	6.603	1.734	25.148	.006
Associated Common Bile Duct Stones (confirmed by EUS or MRI No Yes (ref.)	3.664	2.185	6.145	.000	3.954	1.681	9.300	.002
Gangrenous cholecystitis No Yes (ref.)	2.594	2.006	3.354	.000	1.733	1.086	2.765	.021
Pericholecystic abscess No Yes (ref.)	3.918	2.743	5.595	.000	3.001	1.501	5.998	.002
Hepatic abscess No Yes (ref.)	9.780	3.739	25.582	.000	–	–	–	–
Biliary peritonitis No Yes (ref.)	3.545	1.983	6.338	.000	–	–	–	–
ACC grade 1 2 3 (ref.)	.443 .000 –	.340 .000 –	.578 – –	.000 .999 –	–	–	–	–
*Operative time in min	.988	.985	.991	.000	.994	.990	.998	.007
Bail out procedure? No Yes (ref.)	6.674	4.194	10.621	.000	–	–	–	–
Chole-Risk score 0 (ref.) 1 2 3 4	.715 .483 .151 .039	.505 .333 .089 .005	1.012 .701 .258 .315	.000 – .058 .000 .000 .002	–	–	–	–
POSSUM Operative severity score < 15 > 15 (ref)	12.137	5.434	27.110	.000	8.453	2.073	34.462	.003

*DM* diabetes mellitus, *BMI* Body mass index, *EUS* endoscopic ultrasound, *MRI* magnetic resonance, *ACC* Acute cholecystitis

In the multivariate logistic regression, the independent risk factors for achieving TOUEC were pulse (no tachycardia), low total score on the POSSUM scale, the absence of hypertension, creatinine < 2 mg/dL, the absence of oliguria, short operative time, absence of palpable mass in right upper quadrant, absence of gangrenous cholecystitis, no perivascular abscess, low ASA score, no prior percutaneous cholecystostomy, absence of choledocholithiasis confirmed by EUS or MRI, low POSSUM physiological score and POSSUM Operative Severity Score < 15. Patients who met these parameters were the most likely to achieve TOUEC.

## Discussion

Textbook outcome (TO) is a multidimensional measure for managing the quality of surgical procedures. It allows comparisons between groups and is easy to interpret. One of the main criticisms of TO is that it is an “all or nothing” indicator. Nevertheless, it is a useful tool that has proven to be an independent indicator of survival in the field of cancer surgery. Obviously, patients with TO represent lower costs for the health system [11, 13, 18, 28, 29]. Information on the use of TO in benign pathology is limited.

In our multicenter prospective series of 1246 early urgent cholecystectomies for ACC, 63.3% of patients achieved TOUEC. Due to the practically non-existent literature on TO in gallstones and the absence of internationally accepted parameters for TO in ACC, it is difficult to compare our results with those of other series. We used the following criteria for defining TOUEC: no 30-day mortality, no 30-day postoperative complications (any CD ≥ I was considered non-TO), no readmission within 30 days, and hospital stay ≤ 7 days (75th percentile) and full laparoscopic surgery. In our definition of TOEUC we did not consider reinterventions, since these are performed in patients classified as Clavien-Dindo IIIb and were thus already included; nor did we consider intraoperative complications since their presence tends to be associated with a higher complication rate in the postoperative period and longer hospital stay. The gold standard for cholecystectomy is the laparoscopic approach, and so we believe it is important that this parameter be included in TO, excluding conversions and open cholecystectomies. Unlike Lucocq et al. in their series of elective cholecystectomies [17], we did not exclude subtotal cholecystectomy since it is a resource used in ACC.

The only studies available at present are two single-center retrospective series. In the study by Lucocq et al. just mentioned, a TO rate of 85.5% was obtained in 2166 patients undergoing elective cholecystectomies, and Iseda et al. reported a rate of 81.5% in their study of 189 patients with ACC [17, 18]. We believe the better results recorded in those series are due to the fact that our TO criteria were stricter. In our definition of TOUEC we included only patients with no complications (CD = 0), while both Lucocq and Iseda included patients with CD ≤ 2 [17, 18].

 If we had included minor complications, we would have obtained a TOUEC of 90.1%, even though our series included patients undergoing emergency surgery. In our view, in cholecystectomy, the ideal postoperative period is one without complications. Furthermore, among their criteria Iseda et al. included a non-prolonged stay of ≥ 10 days, without specifying the reason for using this cut-off; in our case, in accordance with the most widely accepted definition of prolonged stay in TO [9] we considered a period of ≥ 7 days (75th percentile of the stay in our series). Mortality in our series was 1.1% higher than that reported by Lucocq et al. (0.3%), while Iseda et al. recorded zero mortality; our increased rate is probably attributable to the multicenter nature of our study in emergency surgical procedures.

Analysing the factors that influence the attainment of TOUEC in our study, we found numerous significant differences in the univariate regression. In Iseda et al.’s study, age > 70 years, hemoglobin < 11.9g/dL and leukocytosis > 18,000/µL were the only independent factors associated with failure to achieve TOUEC. In our series, age and analytical data were significant in the univariate analysis, but not in the multivariate study. The same was the case of bilirubin, INR and platelets. Other studies have established a direct relationship between bilirubin levels > 2 gr/dl and the degree of difficulty of the cholecystectomy, which may be related to the failure to achieve TO [26]. The only analytical parameter in our multivariate study associated with an increased risk of failure to attain TOUEC was creatinine > 2mg/dL.

In our series, probably due to the numerous variables recorded and the large sample size, the POSSUM physiological score, total POSSUM, and ASA all reached significance. The ASA score also independently influenced the achievement of TO in the scheduled cholecystectomies in Lucocq et al.’s study [17]. These data are in line with other published works which show that the higher the risk predictor scores, the higher the rates of morbidity and mortality, length of stay and readmission and that, as a result, the postoperative period is likely to be suboptimal [30–37]. Clinical variables such as tachycardia, pharmacologically treated hypertension, the presence of a palpable mass in the right hypochondrium and the presence of oliguria at diagnosis also reduce the likelihood of achieving TOUEC.

Forms of complicated cholecystitis such as abscesses, choledocholithiasis confirmed by EUS, gangrenous cholecystitis, and perivesicular abscesses were also identified as risk factors for the failure to achieve TOUEC. Previous percutaneous cholecystostomy also had a negative influence, although cholecystostomy has been widely used since the publication of the Tokyo Guidelines, numerous publications have noted its high associated morbidity, the difficulty of the laparoscopic approach, prolongation of hospital stay, and the high readmission rate. As a result, in spite of its value for managing acute episodes in fragile and high-risk patients, it should not be considered as innocuous, or as the gold standard treatment [38–46].

The operative time in our case was a decisive factor in attaining TOUEC: the shorter the postoperative time, the more likely TOUEC was to be achieved. This finding has already been reported in other articles which have demonstrated that prolonged surgical time increases the risk of surgical wound infection and the risk of pulmonary complications, and therefore also increases morbidity and mortality rates and lengthens hospital stay.

The main limitation of the study is the scarcity of literature on the topic and the lack of an internationally accepted definition of TO, which means that it difficult to make comparisons with other series and may have introduced certain biases in the collection of data. As its main strength, this is the first prospective multicenter study that analyses TO in cholecystectomy for acute cholecystitis.

## Conclusion

Among modifiable factors, avoiding unnecessary percutaneous cholecystostomies, using a laparoscopic approach, and keeping surgical time as short as possible are all crucial for achieving TOUEC. Although the other independent factors are probably not modifiable, a rapid optimization of patients with acute cholecystitis is likely to improve postoperative outcomes. To our knowledge, this is the largest prospective series of TO in urgent cholecystectomy published to date. There is a clear need for an international consensus definition of the parameters that the TOUEC should include. Our proposal is: no 30-day mortality, no 30-day postoperative complications (any CD ≥ I is considered non-TO), no readmission within 30 days, and hospital stay ≤ 7 days (75th percentile) and full laparoscopic surgery.

## Data Availability

The datasets generated and/or analyzed during the current study are not publicly available but are available from the corresponding author on reason- able request.

## Abbreviations

ACC: Acute cholecystitis
WSES: World society of emergency surgery
ELC: Early laparoscopic cholecystectomy
LOS: Post-operative length of stay
TO: Textbook outcome
UEC: Urgent early cholecystectomy
TOUEC: Textbook outcome in urgent early cholecystectomy

## References

[1] Wakabayashi G, Iwashita Y, Hibi T, Takada T, Strasberg SM, Asbun HJ, et al. Tokyo Guidelines 2018: surgical management of acute cholecystitis: safe steps in laparoscopic cholecystectomy for acute cholecystitis (with videos). 2018; Available from: http://www.jshbps.jp/modules/en/index.php?content_id=47 10.1002/jhbp.517 29095575

[2] Kimura Y, Takada T, Kawarada Y, Nimura Y, Hirata K, Sekimoto M, Yoshida M, Mayumi T, Wada K, Miura F, Yasuda H (2007). Definitions, pathophysiology, and epidemiology of acute cholangitis and cholecystitis: Tokyo Guidelines. J Hepato-Biliary-Pancreatic Surg.

[3] Keus F, De Jong JAF, Gooszen HG, Van Laarhoven CJHM (2006). Laparoscopic versus open cholecystectomy for patients with symptomatic cholecystolithiasis. Cochrane Database Syst Rev..

[4] Wiggins T, Markar SR, MacKenzie H, Faiz O, Mukherjee D, Khoo DE, Purkayastha S, Beckingham I, Hanna GB (2019). Optimum timing of emergency cholecystectomy for acute cholecystitis in England: population-based cohort study. Surg Endosc..

[5] Pisano M, Allievi N, Gurusamy K, Borzellino G, Cimbanassi S, Boerna D (2020). World society of emergency surgery updated guidelines for the diagnosis and treatment of acute calculus cholecystitis. World J Emerg Surg.

[6] Yokoe M, Takada T, Strasberg SM, Solomkin JS, Mayumi T, Gomi H (2013). TG13 diagnostic criteria and severity grading of acute cholecystitis (with videos). J Hepatobiliary Pancreat Sci.

[7] Cuschieri A, Dubois F, Mouiel J, Mouret P, Becker H, Buess G (1991). The european experience with laparoscopic cholecystectomy. Am J Surg.

[8] Okamoto K, Suzuki K, Takada T, Strasberg SM, Asbun HJ, Endo I (2018). Tokyo Guidelines 2018: flowchart for the management of acute cholecystitis. J Hepatobiliary Pancreat Sci..

[9] Kolfschoten NE, Kievit J, Gooiker GA, Van Leersum NJ, Snijders HS, Eddes EH (2013). Focusing on desired outcomes of care after colon cancer resections; Hospital variations in “textbook outcome”. Eur J Surg Oncol..

[10] Busweiler LAD, Schouwenburg MG, van Berge Henegouwen MI, Kolfschoten NE, de Jong PC, Rozema T (2017). Textbook outcome as a composite measure in oesophagogastric cancer surgery. Br J Surg.

[11] Aiken T, Abbott DE (2020). Textbook oncologic outcome: a promising summary metric of high-quality care, but are we on the same page?. J Surg Oncol.

[12] Görgec B, Benedetti Cacciaguerra A, Lanari J, Russolillo N, Cipriani F, Aghayan D (2021). Assessment of textbook outcome in laparoscopic and open liver surgery. JAMA Surg.

[13] Kulshrestha S, Bunn C, Patel PM, Sweigert PJ, Eguia E, Pawlik TM (2020). Textbook oncologic outcome is associated with increased overall survival after esophagectomy. Surg (United States).

[14] Carbonell Morote S, Gracia Alegría E, Ruiz de la Cuesta Tapia E, Llopis Torremocha C, Ortiz Sebastián S, Estrada Caballero JL, et al. Textbook outcome en cirugía gástrica oncológica, ¿qué implicaciones tiene sobre la supervivencia? Cirugía Española [Internet]. 2021 Oct [cited 2022 Mar 31]; Available from: https://www.elsevier.es/es-revista-cirugia-espanola-36-avance-resumen-textbook-outcome-cirugia-gastrica-oncologica-S0009739X2100302X

[15] Poelemeijer YQM, Marang-van de Mheen PJ, Wouters MWJM, Nienhuijs SW, Liem RSL (2019). Textbook outcome: an ordered composite measure for quality of bariatric surgery. Obes Surg..

[16] Carbonell-Morote S, Ortiz-Sebastián S, Estrada-Caballero JL, Gracia-Alegria E, de la Cuesta R, Tapia E, Villodre C (2023). Textbook outcome in bariatric surgery: evolution during 15 years in a referral center. J Gastrointest Surg..

[17] Lucocq J, Scollay J, Patil P (2022). Evaluation of textbook outcome as a composite quality measure of elective laparoscopic cholecystectomy. JAMA Netw Open..

[18] Iseda N, Iguchi T, Itoh S, Sasaki S, Honboh T, Yoshizumi T (2023). Textbook outcome in the laparoscopic cholecystectomy of acute cholecystitis. Asian J Endosc Surg..

[19] Fugazzola P, Cobianchi L, Di Martino M, Tomasoni M, Dal Mas F, Abu-Zidan FM (2023). Prediction of morbidity and mortality after early cholecystectomy for acute calculous cholecystitis: results of the S. P. Ri. M. A. C. C. study. World J Emerg Surg.

[20] Copeland GP, Jones D, Walters M (1991). POSSUM: a scoring system for surgical audit. Br J Surg.

[21] Prytherch DR, Whiteley MS, Higgins B, Weaver PC, Prout WG, Powell SJ (1998). POSSUM and Portsmouth POSSUM for predicting mortality. Br J Surg.

[22] apache2.pdf

[23] Novotny V, Froehner M, Koch R, Zastrow S, Heberling U, Leike S (2016). Age, American society of anesthesiologists physical status classification and Charlson score are independent predictors of 90-day mortality after radical cystectomy. World J Urol.

[24] Charlson M, Szatrowski TP, Peterson J, Gold J (1994). Validation of a combined comorbidity index. J Clin Epidemiol..

[25] Velanovich V, Antoine H, Swartz A, Peters D, Rubinfeld I (2013). Accumulating deficits model of frailty and postoperative mortality and morbidity: Its application to a national database. J Surg Res..

[26] Di Martino M, Mora-Guzmán I, Jodra VV, Dehesa AS, García DM, Ruiz RC (2021). How to predict postoperative complications after early laparoscopic cholecystectomy for acute cholecystitis: the chole-risk score. J Gastrointest Surg.

[27] Clavien PA, Barkun J, De Oliveira ML, Vauthey JN, Dindo D, Schulick RD (2009). The clavien-dindo classification of surgical complications: Five-year experience. Ann Surg.

[28] Levy J, Gupta V, Amirazodi E, Allen-Ayodabo C, Jivraj N, Jeong Y (2020). Textbook outcome and survival in patients with gastric cancer. Ann Surg.

[29] Merath K, Chen Q, Bagante F, Beal E, Akgul O, Dillhoff M (2020). Textbook outcomes among medicare patients undergoing hepatopancreatic surgery. Ann Surg.

[30] Lai CPT, Goo TT, Ong MW, Prakash PS, Lim WW, Drakeford PA (2021). A comparison of the P-POSSUM and NELA risk score for patients undergoing emergency laparotomy in Singapore. World J Surg.

[31] Darbyshire AR, Kostakis I, Pucher PH, Prytherch D, Mercer SJ (2022). P-POSSUM and the NELA score overpredict mortality for laparoscopic emergency bowel surgery: an analysis of the NELA database. World J Surg.

[32] Alabbasy MM, Elsisy AAE, Mahmoud A, Alhanafy SS (2023). Comparison between P-POSSUM and NELA risk score for patients undergoing emergency laparotomy in Egyptian patients. BMC Surg.

[33] Shekar N, Debata PK, Debata I, Nair P, Rao LS, Shekar P (2023). Use of POSSUM (physiologic and operative severity score for the study of mortality and morbidity) and portsmouth-POSSUM for surgical assessment in patients undergoing emergency abdominal surgeries. Cureus..

[34] Meyer AC, Eklund H, Hedström M, Modig K (2021). The ASA score predicts infections, cardiovascular complications, and hospital readmissions after hip fracture - A nationwide cohort study. Osteoporos Int.

[35] Tran A, Mai T, El-Haddad J, Lampron J, Yelle J-D, Pagliarello G (2017). Preinjury ASA score as an independent predictor of readmission after major traumatic injury. Trauma Surg acute care open..

[36] Teves J, Holc F, Castro Lalín A, García-Mansilla A, Vildoza SRB (2023). Are frailty scores superior to the ASA score in predicting complications, hospital stay, and readmissions in total knee replacement? A comparative study between octogenarian and septuagenarian patients. Rev Esp Cir Ortop Traumatol..

[37] Kastanis G, Topalidou A, Alpantaki K, Rosiadis M, Balalis K (2016). Is the ASA score in geriatric hip fractures a predictive factor for complications and readmission?. Scientifica (Cairo)..

[38] Chon HK, Park C, Park DE, Kim TH (2021). Efficacy and safety of conversion of percutaneous cholecystostomy to endoscopic transpapillary gallbladder stenting in high-risk surgical patients. Hepatobiliary Pancreat Dis Int.

[39] Sosna J, Copel L, Kane RA, Kruskal JB (2003). Ultrasound-guided percutaneous cholecystostomy: update on technique and clinical applications. Surg Technol Int.

[40] Soleimani M, Mehrabi A, Mood ZA, Fonouni H, Kashfi A, Büchler MW (2007). Partial cholecystectomy as a safe and viable option in the emergency treatment of complex acute cholecystitis: a case series and review of the literature. Am Surg.

[41] Polistina F, Mazzucco C, Coco D, Frego M (2019). Percutaneous cholecystostomy for severe (Tokyo 2013 stage III) acute cholecystitis. Eur J Trauma Emerg Surg.

[42] Noubani M, Sethi I, McCarthy E, Stanley SL, Zhang X, Yang J (2023). The impact of interval cholecystectomy timing after percutaneous transhepatic cholecystostomy on post-operative adverse outcomes. Surg Endosc..

[43] Er S, Berkem H, Özden S, Birben B, Çetinkaya E, Tez M (2020). Clinical course of percutaneous cholecystostomies: a crosssectional study. World J Clin Cases.

[44] Stanek A, Dohan A, Barkun J, Barkun A, Reinhold C, Valenti D (2018). Percutaneous cholecystostomy: a simple bridge to surgery or an alternative option for the management of acute cholecystitis?. Am J Surg.

[45] Anderson JE, Chang DC, Talamini MA (2013). A nationwide examination of outcomes of percutaneous cholecystostomy compared with cholecystectomy for acute cholecystitis, 1998–2010. Surg Endosc.

[46] Kourounis G, Rooke ZC, McGuigan M, Georgiades F (2022). Systematic review and meta-analysis of early vs late interval laparoscopic cholecystectomy following percutaneous cholecystostomy. HPB.

[47] Coccolini F, Catena F, Pisano M, Gheza F, Fagiuoli S, Di Saverio S (2015). Open versus laparoscopic cholecystectomy in acute cholecystitis Systematic review and meta-analysis. Int J Surg..

[48] Tafazal H, Spreadborough P, Zakai D, Shastri-Hurst N, Ayaani S, Hanif M (2018). Laparoscopic cholecystectomy: A prospective cohort study assessing the impact of grade of operating surgeon on operative time and 30-day morbidity. Ann R Coll Surg Engl.

[49] Maqsood H, Buddensick TJ, Patel K, Ferdosi H, Sautter A, Setiawan L (2016). Effect of residents on operative time and complications: focus on laparoscopic cholecystectomy in the community. J Surg Educ.

